# Translational medical bioengineering research of traumatic brain injury among Chinese and American pedestrians caused by vehicle collision based on human body finite element modeling

**DOI:** 10.3389/fneur.2023.1296902

**Published:** 2024-01-25

**Authors:** Lingbo Yan, Chenyu Liu, Xiaoming Zhu, Dayong Zhou, Xiaojiang Lv, Xuyuan Kuang

**Affiliations:** ^1^State Key Laboratory of Advanced Design and Manufacture for Vehicle Body, Hunan University, Changsha, China; ^2^Shanghai Motor Vehicle Inspection Certification and Tech Innovation Center Co., Ltd., Shanghai, China; ^3^Geely Automobile Research Institute (Ningbo) Co., Ltd., Zhejiang Key Laboratory of Automobile Safety Technology, Ningbo, China; ^4^Xiangya Hospital, Central South University, Jiangxi, National Regional Center for Neurological Diseases, Nanchang, Jiangxi, China; ^5^Department of Hyperbaric Oxygen, Xiangya Hospital, Central South University, Changsha, Hunan, China; ^6^National Clinical Research Center for Geriatric Disorders (Xiangya), Changsha, Hunan, China

**Keywords:** traumatic brain injury, medical bioengineering, finite element simulation, pedestrian protection, translational and engineering research

## Abstract

Based on the average human body size in China and the THUMS AM50 finite element model of the human body, the Kriging interpolation algorithm was used to model the Chinese 50th percentile human body, and the biological fidelity of the model was verified. We built three different types of passenger vehicle models, namely, sedan, sports utility vehicle (SUV), and multi-purpose vehicle (MPV), and used mechanical response analysis and finite element simulation to compare and analyze the dynamic differences and head injury differences between the Chinese 50th percentile human body and the THUMS AM50 model during passenger vehicle collisions. The results showed that there are obvious differences between the Chinese mannequin and THUMS in terms of collision time, collision position, invasion speed, and angle. When a sedan collided with the mannequins, the skull damage to the Chinese human body model was more severe, and when a sedan or SUV collided, the brain damage to the Chinese human body was more severe. The abovementioned results suggest that the existing C-NCAP pedestrian protection testing regulations may not provide the best protection for Chinese human bodies, and that the regulations need to be improved by combining collision damage mechanisms and the physical characteristics of Chinese pedestrians. This thorough investigation is positioned to shed light on the fundamental biomechanics and injury mechanisms at play. Furthermore, the amalgamation of clinically rooted translational and engineering research in the realm of traumatic brain injury has the potential to establish a solid foundation for discerning preventive methodologies. Ultimately, this endeavor holds the potential to introduce effective strategies aimed at preventing and safeguarding against traumatic brain injuries.

## Introduction

Pedestrian accidents have always accounted for a significant proportion of traffic accidents, and have a high incidence of serious injuries and mortality rates. In Europe, 23% of traffic accident fatalities are pedestrians, whereas pedestrians account for 11% of fatalities in traffic accidents in the United States, and that number increases to over 25% in China (1). This means that on average, in China, nearly 25,000 pedestrians die in traffic accidents every year, ranking first among all traffic accidents in the country. Head injury is the main cause of pedestrian death, accounting for approximately 54% of pedestrian traffic accident fatalities (2). Head injury is mainly divided into skull fracture and brain injury, with brain injury being divided into focal brain injury (hematoma, contusion, etc.) and diffuse brain injury (diffuse axonal injury, concussion, etc.).

In recent years, to acquire a better understanding of the mechanisms of injury to pedestrians’ skulls and brains, many researchers have developed finite element models of the human head and have used finite element simulations to simulate collisions between passenger vehicles and pedestrians. Watanabe et al. (3) established an SUV pedestrian collision simulation using the THUMS pedestrian finite element model and studied the impact of collision speed on pedestrian head and chest injuries. Tamura et al. (4) developed a finite element model of the brain representing a 50th percentile male and combined it with the THUMS model to explore the mechanism of pedestrian head injury through simulation. Yang et al. (5) and others from Hunan University established a head finite element model containing muscles, spinal cord, and complete brain structure. Wei (6) combined it with the LSTC Hybrid—III finite element model to analyze the impact of different vehicle speeds and pedestrian gait on brain injury.

**Figure 1:**
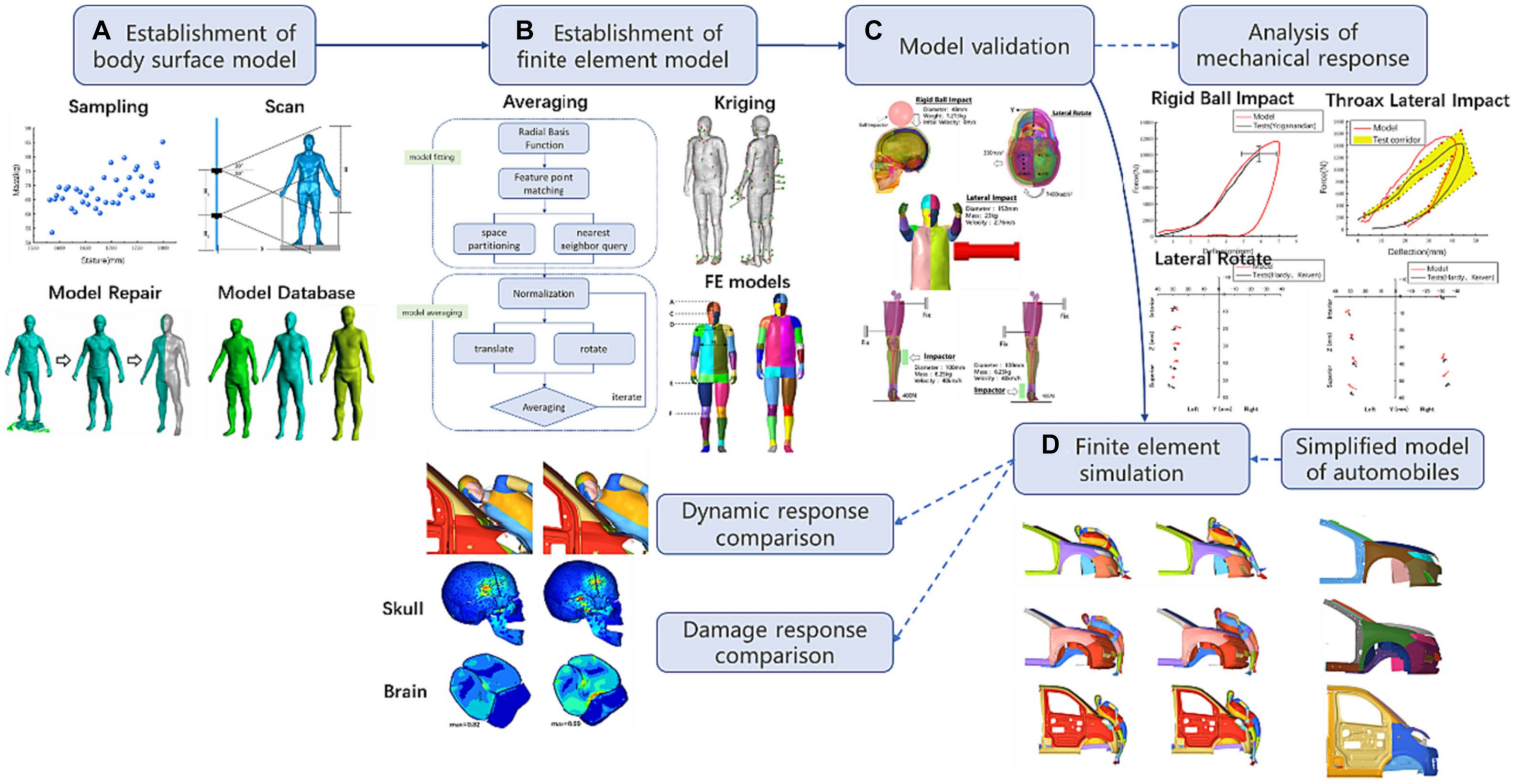
The workflow and technology roadmap. **(A)** The process of establishing a database of Chinese male body surface models, **(B)** the process of averaging body surface models and the method of establishing a finite element model of the Chinese 50th percentile male body, **(C)** the simulation verification of the biological fidelity of the finite element model of Chinese 50th percentile human body, and **(D)** the construction of vehicle-pedestrian simulation conditions and the analysis of simulation results.

However, the abovementioned pedestrian collision damage studies were conducted using THUMS human finite element models or Hybrid III dummy finite element models representing European and American anthropomorphic characteristics. There are certain differences in the average human body size between Europe, America, and China, with differences in length, mass, and center of gravity of each body segment leading to differences in pedestrian movement trajectory, head collision angle, position, and degree of injury under the same working conditions, leading to different design goals for pedestrian protection structures. Many countries and institutions have established three-dimensional human body shape measurement databases through optical scanning to obtain more detailed human body data, such as the United Kingdom three-dimensional human body size database in the United Kingdom (7) and the CAESAR database established in North America, the Netherlands, and Italy (8). However, there is currently no authoritative Chinese human body shape measurement database available. Therefore, this article establishes a Chinese standing human body surface model through optical scanning and uses the grid transformation method to establish a finite element model of the Chinese 50th percentile male body. By comparing the impact simulation results of various parts of the body with literature data, their biological fidelity was verified. The mechanism of skull and brain damage in pedestrian collision accidents was analyzed in detail through simulation, and the dynamic response and head injury differences between Chinese and American pedestrians in different passenger vehicle models were compared. The workflow is shown in Figure 1.

## Materials and methods

### Chinese body finite element modeling

According to the latest statistical results of the Chinese Academy of Standardization on the basic ergonomic parameters of Chinese adults from 2014 to 2019 (9), using the Latin square sampling method, 40 adult male volunteers who met anthropometric and health standards and had no physiological diseases, such as scoliosis, were uniformly selected for three-dimensional optical scanning to generate a body surface model. Reverse engineering software was used to process the initial body surface model with hole repair, surface smoothing, symmetry, etc., and finally obtain the Chinese male body surface model data randomly distributed between the 5 and the 95th percentile.

In anthropometry, the 5, 50, and 95th percentiles are most commonly used, representing small, medium, and large people, respectively. In this study, the 50th percentile male was taken as the representative to compare the difference between Chinese and American human body injuries caused by vehicle collisions. The THUMS AM50 standing human finite element model (version 4.02) was selected to simulate a 50th percentile male human body in Europe and America. This model was developed in collaboration with Toyota Motor Corporation and Toyota Central R&D Labs for pedestrian collision safety research. The model is 1,786 mm tall, weighs 77.6 kg, and has a complete skeletal structure and internal organs. Its biological fidelity has been fully verified (10, 11). The finite element model of Chinese pedestrians was obtained through grid transformation based on the THUMS model and the Chinese 50th percentile human body surface model. Three male volunteers from Henan, Anhui, and Hainan, aged between 22 and 25 years old, whose height and weight were close to the 50th percentile, were selected from the database for average processing to build up an accurate Chinese 50th percentile body surface model. The average process is shown in Figure 1. Averaging the data of the Chinese 50th percentile body surface model, the Kriging interpolation algorithm was used to perform grid transformation on the THUMS pedestrian finite element model and generate the Chinese 50th percentile finite element model.

### Verification of actual damage of human finite element model in lateral impact

When a vehicle impacts a pedestrian laterally, the most vulnerable body parts are the head, chest, and lower limbs. Therefore, the injury responses of the Chinese mannequin are tested in the head, chest, and lower limbs parts. See Table 1 for the list and comparison with the simulation results.

**Table 1:** Validation list of the chinese mannequin position.

	Simulation settings	Mechanical response comparison
Head	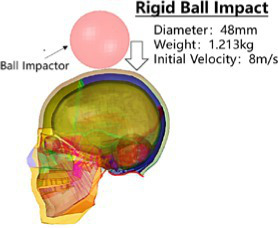	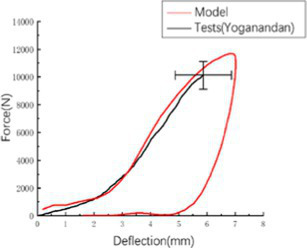
	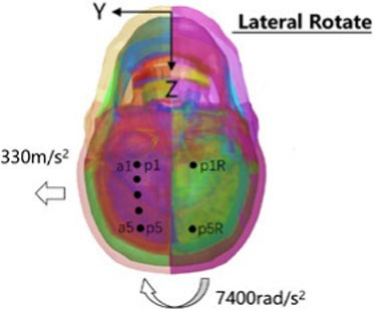	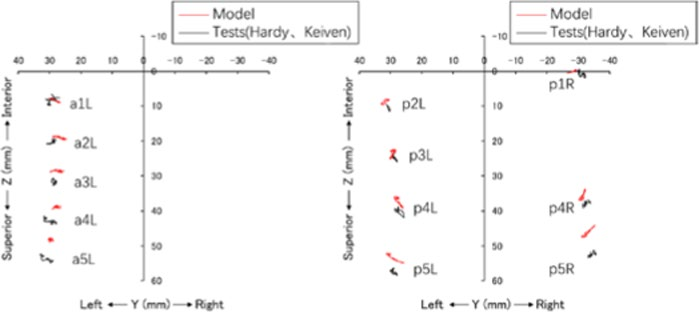
Chest	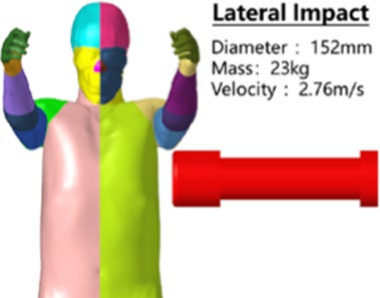	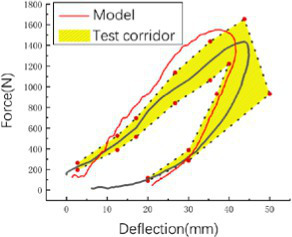
Lower limb	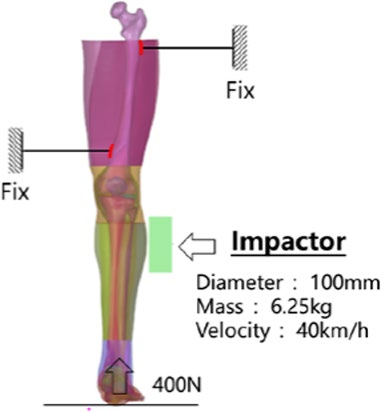	① Bending moment at initial damage moment Test: 369–545 Nm, Simulation: 356 Nm
		② Shear force at initial damage time Test: 1.6–2.3KN, Simulation: 2.5KN
		③ Knee bending angle Test: 1.7–4.1°, Simulation: 5°
		④ Knee shear displacement Test: 13–14 mm, Simulation: 16 mm
	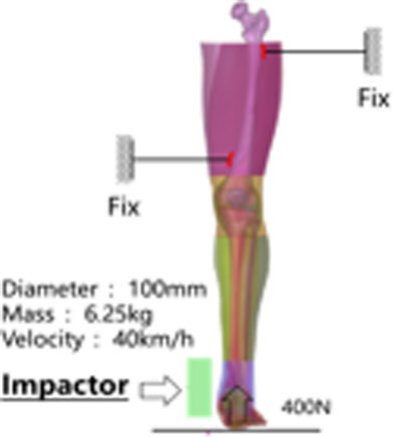	①Bending moment at initial damage moment Test: 367–450 Nm, Simulation: 406 Nm
		② Shear force at initial damage time Test: 1.2–1.4KN, Simulation: 2.9KN
		③ Knee bending angle Test: 10–14.8°, Simulation: 16°
		④ Knee shear displacement Test: 13–17 mm, Simulation: 30 mm

A rigid ball impactor with a mass of 1.213 kg and a radius of 48 mm was used to impact the overhead area at an initial speed of 8.0 m/s, simulating the head rigid ball impact test conducted by Yoganandan et al. (12), while fixing the skull base, and recording the force-displacement curve of the impactor. The results of test marks 7–12 were selected as the verification basis for the head impact simulation of the Chinese Mannequin (see Supplementary Material 1).

The head rotated in the side impact direction with 7,400 rad/s2 angular acceleration, simulating the head rotation test of Hardy and Kleiven (13, 14), and we recorded the *y* and *z* direction displacement of each marker point of the brain relative to the skull. Using a cylindrical impactor with a mass of 23 kg and a diameter of 152 mm, we simulated the lateral impact test of Shaw et al. (15) on the human chest at a speed of 2.76 m/s, and recorded the force deformation response curve of the chest.

We fixed the proximal and distal ends of the femur with screws and set a fixation plate near the knee joint at the distal end of the femur to limit the movement of the femur. Then, a preload of 400 N was applied axially from the foot to the tibia, simulating the gravity of the upper body when standing in a human posture. In the shear test, the impactor impacted the lower part of the knee joint from the outside of the leg at a speed of 40 km/h, and in the bending test, the impactor impacted the ankle joint from the inside of the leg at a speed of 40 km/h. The impactor mass of both tests was 6.25 kg, and the impact surface was connected with a 50 mm thick foam material to simulate the bending and shear test of the lower limbs of Kajzer (16, 17). In Kajzer’s experiment, the cadaver samples numbered 11B, 12S, 13S, and 14B were all male (see Supplementary Material 2), with an average height and weight of 169 cm and 68 kg, which are similar in size to the Chinese 50th percentile male human body. Therefore, the results of experiments numbered 12S and 13S were selected as controls for shear simulation, and the results of experiments numbered 11B and 14B were selected as controls for bending simulation. The knee bending angle, knee joint bending moment, and initial injury time were compared to Shear displacement and other data.

#### Statistical method

Considering the significant differences in size between cadaver samples and the inability to fully represent the 50th percentile standard human body, the author used the Mertz Viano (18, 19) method to standardize the chest mechanical response curve obtained from the test to generate a chest standard mechanical response curve representing the 50th percentile Chinese human body under a lateral impact (see Supplementary Material 3), and developed a standard response interval using the method of Lobdell (20).

### Vehicle modeling with three different basic structures and collision simulation with different mannequins

According to the pedestrian traffic accident database and various pedestrian accident investigation reports (21–23), passenger vehicles account for the highest proportion of pedestrian accidents. Therefore, three different structural shapes, namely, sedans, SUVs, and MPVs, were selected to construct a finite element model of passenger vehicles. The simplified model retains the structure of the A-pillar, windshield, bumper, crash beam, hood, engine, battery, etc., and is positioned at the center of gravity-defined centralized mass. The initial models of the three vehicle models have been validated (24–26).

We performed a simulation using the finite element analysis program LS-DYNATM V971. In the simulation, a speed of 40 km/h was applied to each vehicle model to collide with the Chinese mannequin and the THUMS model. Then, we output the resultant head acceleration of two mannequins, Head Injury Criterion (HIC15) and Brain Injury Criteria (BRIC), with equivalent stress to the skull and maximum principal strain to the brain. Based on the simulation results of different models in China and the United States in collisions with three different types of passenger vehicles, suggestions are given for the C-NCAP pedestrian protection testing procedure. To avoid the randomness of the conclusion, pedestrian collision simulations at speeds of 30 and 50 km/h were conducted under the same conditions to further validate the conclusion.

## Results

### Construction and validation of a finite element model of the Chinese human body

**Figure 2:**
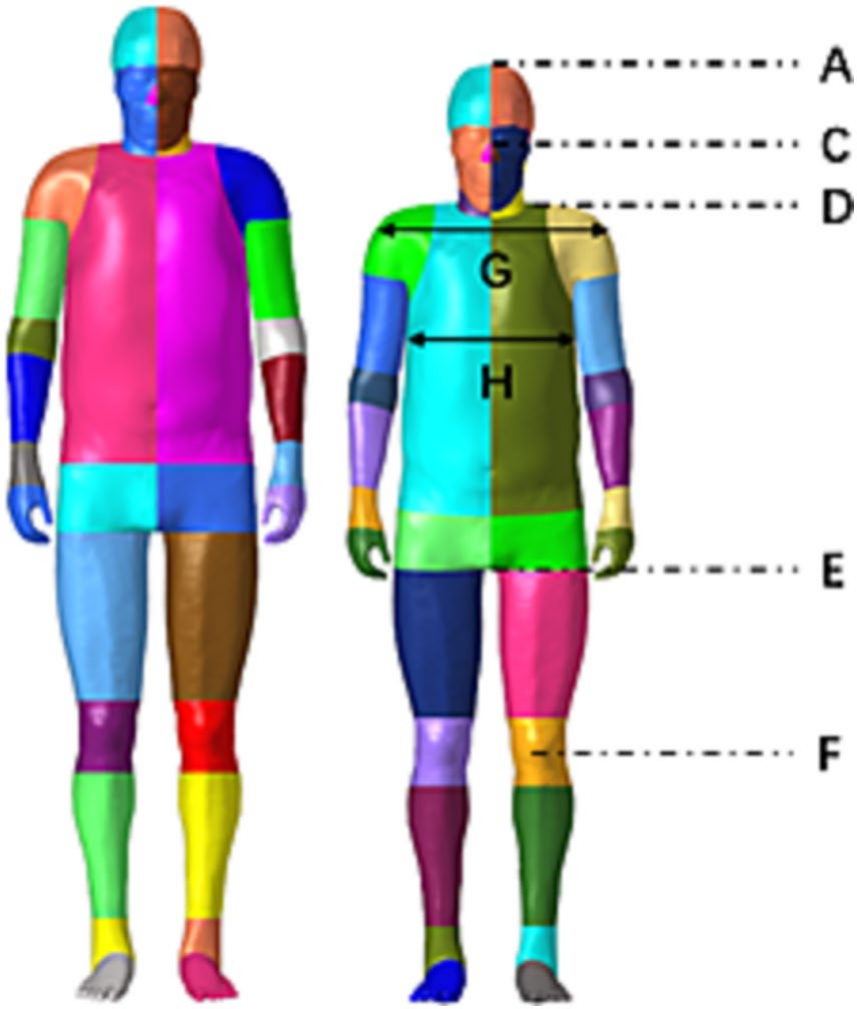
Comparison between THUMS and the Chinese Human Body Model (L: THUMS Model, R: Chinese Human Body Model).

The comparison between the Chinese and American mannequins is shown in Figure 2, and the size comparison data are shown in Table 2. The Chinese mannequin is smaller than the THUMS mannequin in size, shoulder width, and chest thickness, and the position of the pelvis, femur, and tibia is also lower. Comparing the output results of various verification simulations with the test results, it was observed that the simulation results were basically within the range of the test results, so the biological fidelity of the Chinese mannequin could be verified.

**Table 2:** Comparison of human body model dimensional data.

Size	Chinese 50th percentile body size (male)	Chinese mannequin	THUMS
A. Height	1,690 mm	1,689 mm	1,786 mm
B. Weight	67.6 kg	67.3 kg	77.6 kg
C. Eye level	1,568 mm	1,560 mm	1,659 mm
D. Shoulder height	1,387 mm	1,367 mm	1,481 mm
E. Perineal height	790 mm	790.7 mm	826.4 mm
F. Tibial height	444 mm	442.7 mm	460.1 mm
G. Shoulder width	431 mm	434 mm	446 mm
H. Thorax width	280 mm	288 mm	312 mm

### Vehicle structure simulation and collision simulation

**Figure 3:**
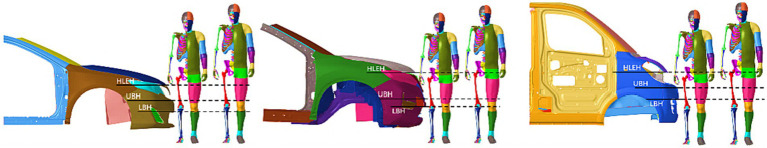
Comparison of vehicle structure and relative position of human body (L: sedan, Center: SUV, R: MPV).

For the test, we maintained two human body models in a standing posture, positioned at the front of the vehicle along the middle line of the vehicle, with their orientation perpendicular to the direction of vehicle travel. Figure 3 shows the relative positions of the front-end shapes of the three vehicle models and various parts of the human body at the initial moment.

#### Illustration

When the sedan model and the mannequins collided, the head of the Chinese mannequin came into contact with the vehicle at 118 ms with an invasion angle of 81°. The head of the THUMS model came into contact with the vehicle at 128 ms with an invasion angle of 58°. When the SUV model and the mannequins collided, the head of the Chinese mannequin came into contact with the vehicle at 94 ms and the intrusion angle was 87°, whereas the head of the THUMS model came into contact with the vehicle at 104 ms with an intrusion angle of 87°. When the MPV model and the mannequins collided, the head of the Chinese mannequin came into contact with the vehicle at 90 ms with an invasion angle of 51°, and the head of the THUMS model came into contact with the vehicle at 96 ms with an invasion angle of 40°.

**Figure 4:**
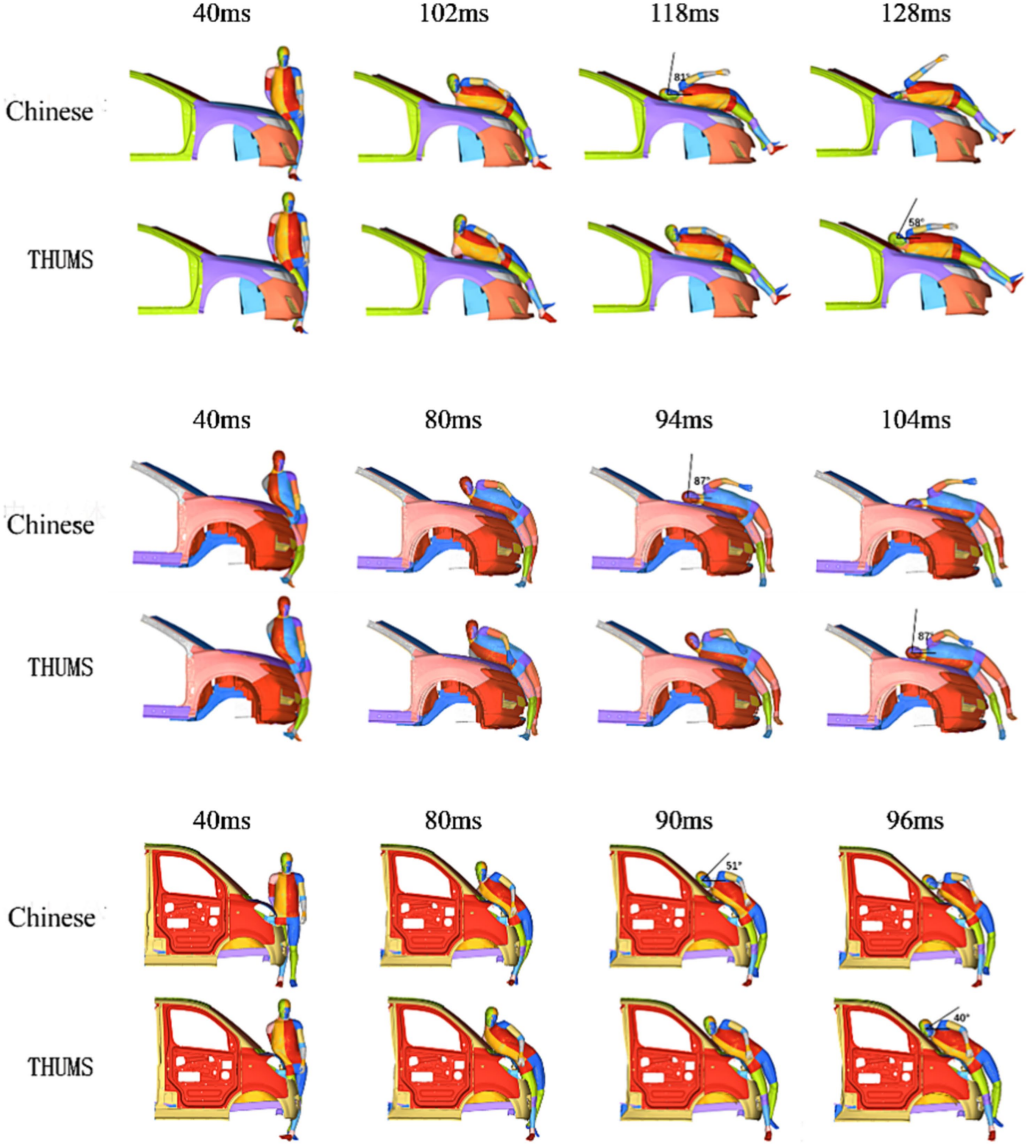
Comparison of kinematics responses of mannequins in different countries during side collision.

**Figure 5:**
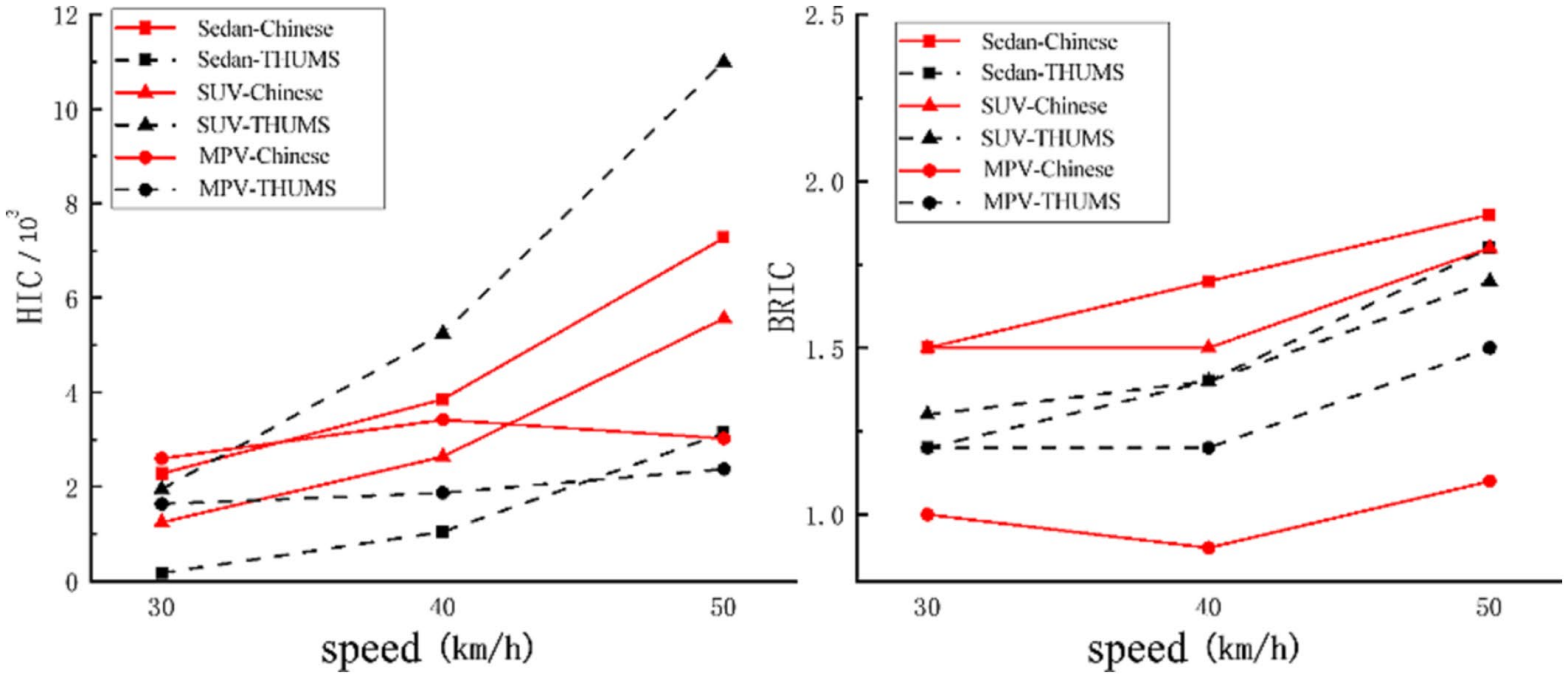
Comparison of HIC and BRIC of mannequin heads at different speeds.

Figure 4 shows the trajectory of pedestrians during the collision process of three vehicle models at a speed of 40 km/h. The lower limbs first came into contact with the bumper and accelerated forward under the impact of the vehicle. The upper body rotated in the direction of the vehicle hood due to inertia, causing the buttocks, abdomen, chest, and shoulders to collide with the hood or windshield in sequence. Finally, the head rotated toward the engine hood or windshield for impact. There are three main damage mechanisms in head-to-vehicle collisions: concentrated compressive force, viscous load in the skull, and inertial load in the brain. Concentrated compressive force may lead to skull fractures, and the inertial load during rotation will cause relative motion between the skull and the brain, resulting in a high strain that may cause brain damage. When the same vehicle model collided with two types of human bodies at the same speed, compared to the THUMS, the rotation radius of the buttocks, abdomen, chest, and head of the Chinese human body was smaller, the contact time with the vehicle came earlier, the entire collision process was shorter, and the contact position was also closer to the front. When the sedan collided with the mannequins, the Chinese human head collided with the rear end of the hood and the transition area of the windshield at 118 ms, with intrusion speeds and angles of 81°and 40.4 km/h, respectively, while the THUMS head collided with the windshield at 128 ms, with intrusion speeds and angles of 58°and 39.9 km/h, respectively. When the SUV model collided with the mannequins, due to the high hood, the Chinese human head collided with the hood at 94 ms, and the THUMS head collided with the high stiffness area at the rear of the hood at 104 ms. Both types of human heads had intrusion angles of 87°. The velocity of the Chinese mannequin’s head was 40.8 km/h, while the THUMS model’s head velocity was 41.6 km/h. When encountering an MPV collision, the rotation amplitude of the pedestrian’s chest was smaller, resulting in earlier head-to-vehicle collisions at lower speeds and angles. The Chinese human head collided with the MPV windshield at a speed and angle of 23.4 km/h and 51°, respectively, at 90 ms, while the THUMS head collided with the windshield at a speed and angle of 23.8 km/h and 40°, respectively, at 96 ms. The collision simulation at different speeds is shown in Figure 5. The results show that although the change in vehicle speed led to changes in the movement trend and injury response of each mannequin, the comparison results of skull injury and brain injury were consistent at 40 km/h.

**Figure 6:**
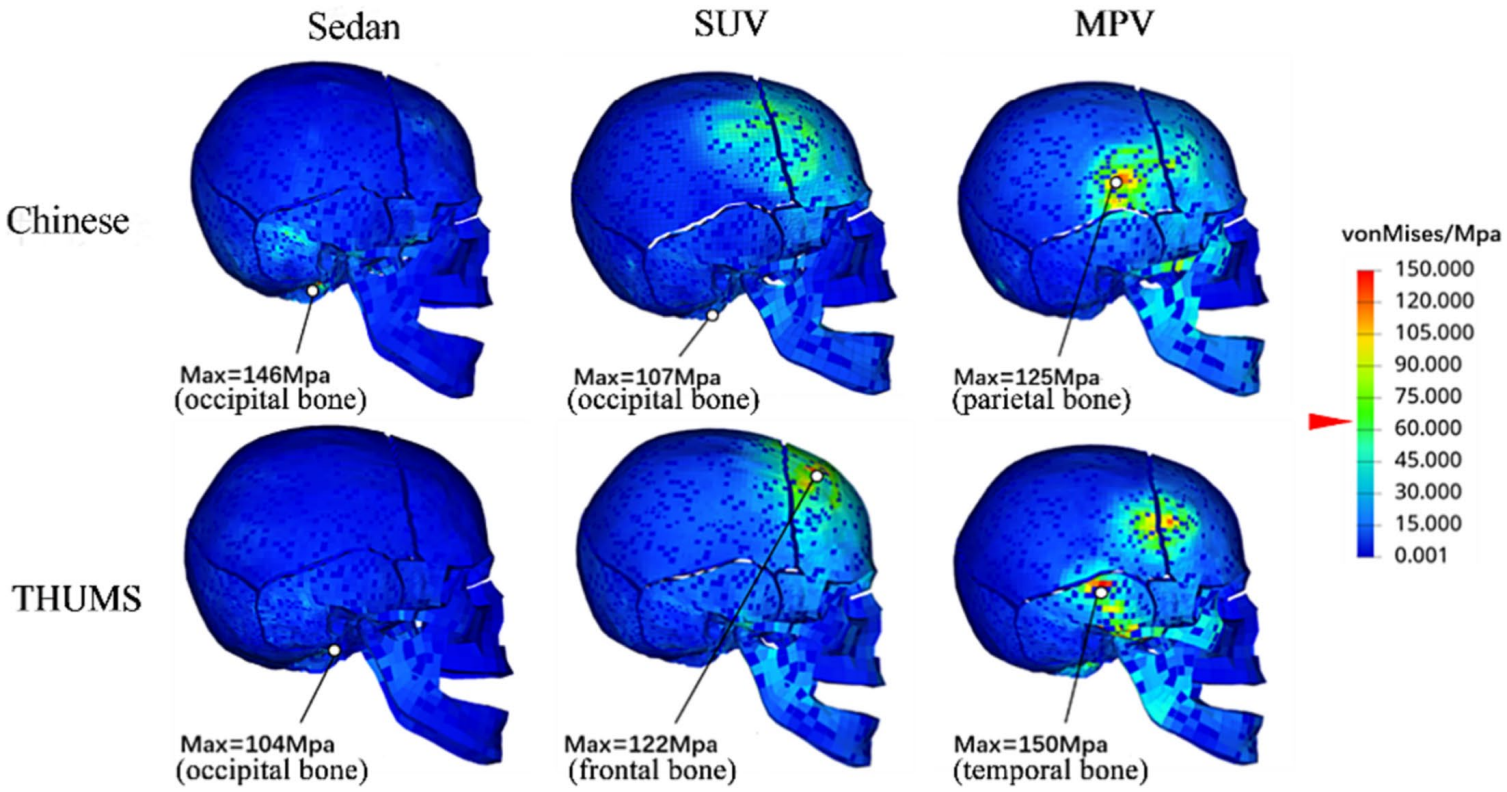
Cloud diagram of equivalent force on the collision side of the skull.

**Figure 7:**
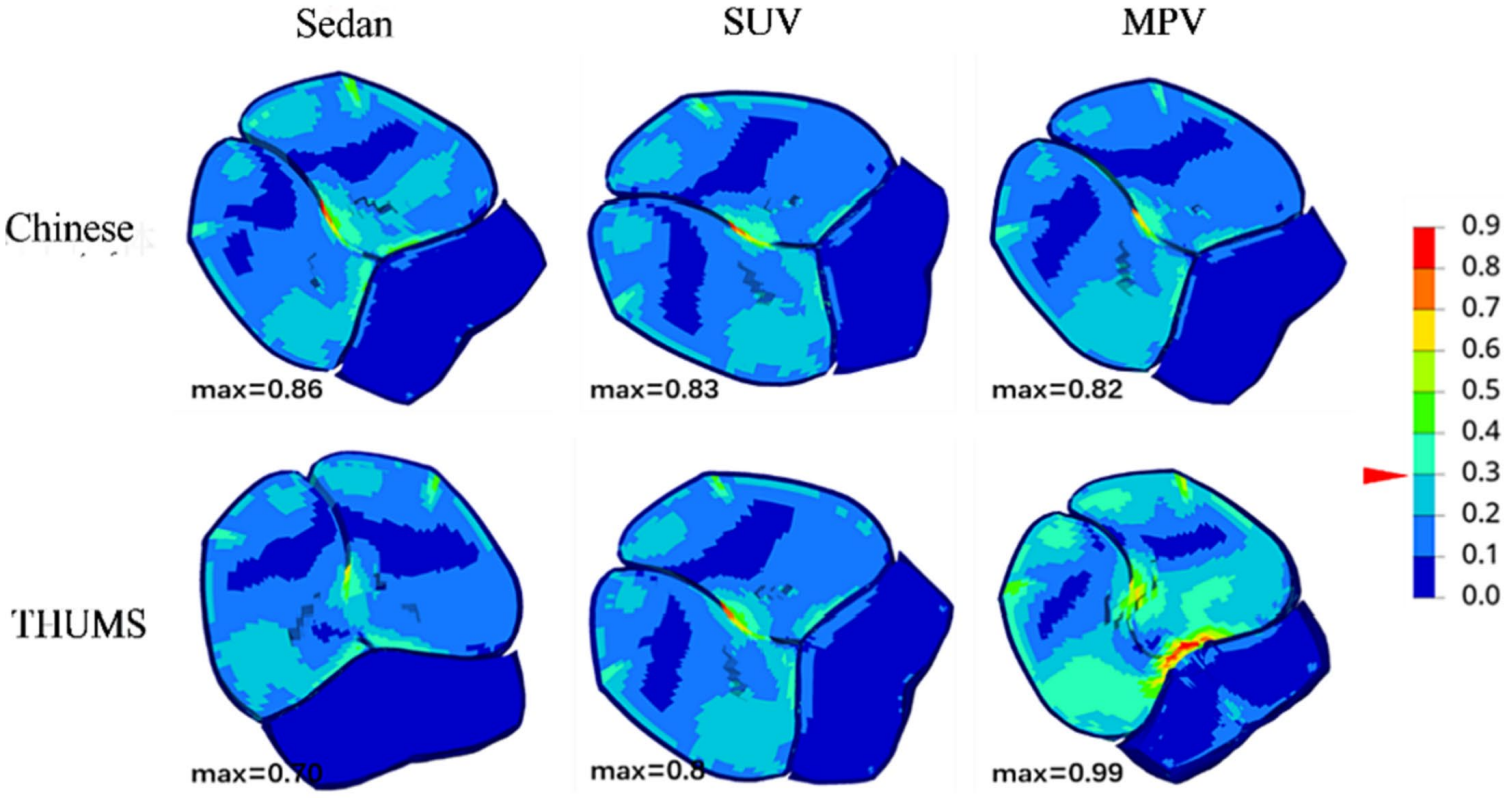
Principal strain cloud map of the central cross-section of the brain.

Table 3 shows the peak response of two types of human head injuries in collisions for each vehicle type at a speed of 40 km/h, with all HIC values higher than the injury standard value of 700. Figure 6 shows the equivalent force cloud map of the skull collision side and the differences in collision position and angle result in different stress distributions in two types of human skulls. Figure 7 shows the principal strain cloud map of the central cross-section of the brain at the moment of maximum principal strain (MPS).

**Table 3:** Comparison of head injury response peaks (vehicle speed 40 km/h).

	Human model	Sedan	SUV	MPV	Reference number
Head integrated acceleration (g)	Chinese human body	196	185	211	/
	THUMS	119	254	139	
HIC_15_	Chinese human body	3,854	2,639	3,419	700
	THUMS	1,046	5,245	1870	
BRIC	Chinese human body	1.7	1.5	0.9	1
	THUMS	1.4	1.4	1.2	
Maximum equivalent stress on the skull (Mpa)	Chinese human body	146	107	125	65
	THUMS	104	122	150	
Brain tissue MPS	Chinese human body	0.86	0.83	0.82	0.3
	THUMS	0.7	0.8	0.99	
Brain tissue CSDM	Chinese human body	0.25	0.67	0.39	0.54
	THUMS	0.08	0.61	0.35	

When the sedan model and mannequins collided, the maximum stress on the Chinese mannequin skull was 146 Mpa, which appeared in the occipital bone, and the maximum stress on the THUMS model skull was 104 Mpa, which appeared in the occipital bone. When the SUV model and mannequins collided, the maximum stress on the Chinese mannequin skull was 107 Mpa, which occurred in the occipital bone, and the maximum stress on the THUMS model skull was 122 Mpa, which occurred in the frontal bone. When the MPV model and mannequins collided, the maximum stress on the Chinese mannequin skull was 125 Mpa, which appeared in the parietal bone, and the maximum stress on the THUMS model skull was 150 Mpa, which appeared in the temporal bone.

The maximum principal strain on the brain of the Chinese mannequin was 0.86, and the maximum principal strain on the brain of the THUMS model was 0.70 when the sedan model crashed. When the SUV model and mannequins collided, the maximum principal strain on the Chinese mannequin brain was 0.83, and the maximum principal strain on the THUMS model brain was 0.80. The maximum principal strain on the brain of the Chinese mannequin was 0.82, and the maximum principal strain on the brain of the THUMS model was 0.99 during the collision of the MPV model.

## Discussion

**Figure 8:**
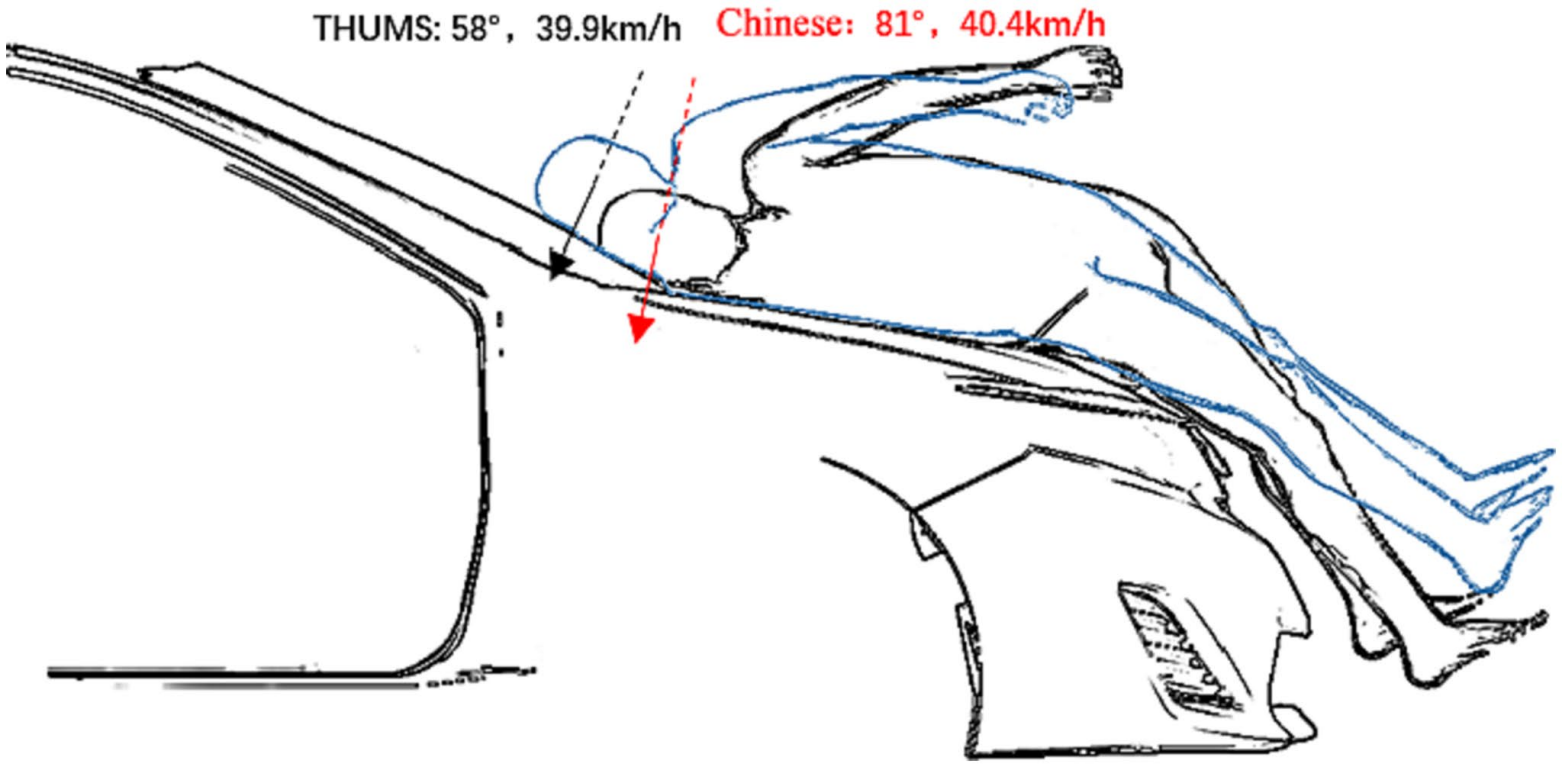
Comparison of head impact velocity and angle between two mannequins.

In the 2021 version of the C-NCAP management rules, the pedestrian protection test specifies that the impact speed of the adult headform impactor is 40 km/h and the impact angle is 60 °(windshield) (27). The present paper simulated the kinematics response of the head when a sedan impacted two mannequins at a speed of 40 km/h (Figure 8). When the head came into contact with the vehicle, the speed of the THUMS head center of gravity was 39.9 km/h, and the angle was 58°. The speed of the Chinese head center of gravity was 40.4 km/h, and the angle was 81°, much higher than the 60° set in the test. The larger impact angle of the Chinese human head also led to higher *Z*-axis acceleration, resulting in higher HIC values when impacted by the rear structure of the hood. Therefore, when conducting pedestrian protection head impact tests targeting the Chinese human body, it is possible to consider adjusting the head impact angle, especially at the transition area between the windshield and the rear end of the hood.

The HIC value depends on the head impact speed, the location of the head being impacted, and the contact area with the vehicle body. When the sedan model collided with the mannequins at a speed of 40 km/h, the Chinese human head collided with the transition area between the engine hood and windshield, while the THUMS head collided with the windshield. The difference in stiffness between the two areas resulted in a difference in HIC values. The collision situation of the SUV model was the same, with the THUMS head colliding with the rear end of the engine hood. The collision position of the Chinese human head was more forward, with lower structural stiffness, resulting in a lower HIC value. During the MPV collision, the head of the two mannequins collided with the windshield. However, due to the different impact angles, the initial impact parts of the head of the two mannequins were significantly different. The head of the Chinese human body collided with the vehicle first, and the face of the THUMS collided with the vehicle first, which led to a higher HIC of the head of the Chinese human body, and also led to a difference in the damaged parts of the skull of the two human bodies. A previous study (28) also proved that there is a significant positive correlation between pedestrian head collision angle and head linear acceleration.

The collision impact force with vehicle structural components is the main cause of skull fractures. According to the literature (29, 30), the fracture stress of the cortical bone of the skull is between 48 and 128 Mpa, and the fracture stress of the dense bone is between 32 and 74 Mpa. Wood et al. determined that the fracture stress of the skull is approximately 65 Mpa. Using 65 Mpa as the threshold, when the sedan collided with the mannequins, the occipital and sphenoid bones of both types of human bodies fractured. When the SUV collided, fractures occurred in the parietal, frontal, occipital, and sphenoid bones of both types of human bodies. When the MPV collided, all the skulls on the impact side were fractured, and the maximum stress in the Chinese human body was concentrated in the parietal bone, while the maximum stress in the THUMS was concentrated in the temporal bone. We then calculated the fracture area of the skull. When the sedan collided, the fracture area in the Chinese human skull was 1,139 mm^2^, and in the THUMS it was 197 mm^2^. When the SUV collided, the fracture area in the Chinese human skull was 1,690 mm^2^, and in the THUMS it was 3,629 mm^2^. When the MPV collided, the fracture area in the Chinese human skull was 944 mm^2^, and in the THUMS it was 1726 mm^2^.

From this, it can be seen that, both in terms of peak stress and fracture area, the sedan model led to more severe damage to the Chinese human skull, while the SUV and MPV models led to more severe damage to the THUMS skull. It is worth noting that although the MPV model caused more severe damage to the skull of THUMS, the HIC value of THUMS was lower than that of the Chinese human body because it is related to the head impact angle mentioned above. This phenomenon further demonstrates the important role of head impact angle in skull injury analysis and also suggests the limitations of headform impactors in simulating complex skull structures in humans.

By measuring and comparing the velocities and accelerations of two different parts of the human body, it was inferred that the magnitude of head angular velocity was mainly related to chest acceleration. When the shoulders of the mannequin came into contact with the engine hood, the chest speed decreased rapidly. The difference between the head speed and the chest speed led to the rapid increase of the head angular speed. Therefore, when the shoulders came into contact with the structural parts with greater stiffness, the chest decelerated faster and the head BRIC was larger. The inevitable drawback of this study is that the HIC criterion based on linear composite acceleration lacks consideration for other brain injury load conditions. For example, when the head is subjected to inertial loads in the acceleration field, relative motion between the skull and brain will occur, resulting in high shear strain and strain rate that can lead to brain tissue damage, and this type of brain damage is likely to occur without skull fractures. The 2018 version of the US New Car Evaluation Regulations (US-NCAP2018) requires that the BRIC value be calculated from the angular velocities in the three different directions of the head. According to the study by Thakhonts et al. (31), when the BRIC is 1, the probability of brain AIS4+ injury is 50%, and when the BRIC is 1.5, the probability of brain AIS4+ injury reaches 80%. According to this conclusion, the probability of AIS4+ injury in Chinese human bodies during sedan and SUV collisions exceeds 80%, with a higher probability of brain damage. In contrast, during MPV collisions, the THUMS model’s AIS4 + damage probability exceeded 50%, resulting in a higher probability of brain damage. The study conducted by Watanabe et al. (3) also reached similar conclusions.

The study of Bain et al. (32) showed that brain tissue damage and contusion occur when the main strain of the white matter exceeds 30%. According to this standard, all three vehicle models can cause damage to brain tissue when driving at a speed of 40 km/h. For the prediction of diffuse axonal injury (DAI), Thakhonts et al. (31) proposed the cumulative strain damage measurement (CSDM) as an evaluation metric, which assumes that DAI occurs when a principal strain of over 0.25 occurs in 49% of the entire brain region. As shown in Table 3, the head injury volume of the Chinese human body during collisions of the three vehicle models was greater than that of THUMS, and during SUV collisions, both types of human head injuries occurred. In addition, Thakhonts also proposed that there is a 50% probability of AIS4 + level severe brain injury occurring when MPS equals 0.89, so when MPV collides with THUMS, DAI may also occur in the head.

The research results suggest that the peak principal strain of brain tissue occurs 1–5 ms before the collision between the head and the vehicle. At this time, the angular velocity of the brain is accelerating and approaching the peak, indicating that in this study, the MPS of brain tissue is mainly related to the inertial load during the rotation process. When the head collides with the vehicle, the MPS decreases, but the volume of brain tissue with strain exceeding 0.25 sharply increases, indicating that the CSDM value is affected by the combined impact force and rotational inertia load. This not only explains the difference in the comparison of MPS and CSDM values when the MPV model collided with the two types of human bodies but also indicates that when analyzing the strain of pedestrian brain tissue, these two indicators should be considered comprehensively.

Mueller (33) counted the HIC and BRIC values of the Hybrid III dummy in 128 crash tests and found that there was no correlation between HIC and BRIC. They assessed the risk of head injury from different angles and concluded that the two criteria should be used together. Takcounts et al. also provided a similar explanation. In the present article, it could also be observed that when using HIC and BRIC values to compare two types of human head injuries, the comparison results may not be consistent. For example, when the SUV model collided with the two types of human bodies, the HIC value of the Chinese human body was lower than THUMS, but the BRIC value was higher. Based on the injury response of the skull and brain tissue, it could be concluded that the degree of skull injury in Chinese humans was lower than that in THUMS, but the brain tissue injury was more severe. Therefore, it is necessary to combine these two indicators when analyzing pedestrian head injury.

Overall, at a collision speed of 40 km/h, the Chinese human body suffers more severe skull damage in sedan collisions and more severe brain damage in sedan and SUV collisions, whereas THUMS suffers more severe skull damage during SUV and MPV collisions, and more severe brain damage during MPV collisions. When the C-NCAP pedestrian protection testing procedure based on THUMS is applied, it may not provide the best protection for Chinese pedestrians. It is recommended that the testing procedure be improved by combining collision damage mechanisms and the physical characteristics of Chinese pedestrians.

In this research, a comparison was made between the differences in head and brain injuries resulting from vehicle collisions among the 50th percentile adult males in China and the United States. When factors such as pedestrian height, weight, age, initial collision posture, and other variables vary, the outcomes of injuries can vary significantly. Therefore, this research cannot account for variations in pedestrian injuries between the two countries in all circumstances. Furthermore, this study compared differences in bodily injuries between pedestrians of the two countries using the same tolerance threshold. In the future, it is essential to take into account a broader range of body types and conduct more in-depth research based on variations in individual tolerance thresholds.

## Conclusion

This article constructs a finite element model of the Chinese 50th percentile human body and achieves the required biological fidelity. Through finite element simulation, the head dynamics and injury response of the Chinese mannequin and the THUMS model in the collision of sedans, SUVs, and MPVs were compared. It was found that the collision time, collision position, invasion speed, and angle of the Chinese mannequin head were significantly different from those of the THUMS. When a sedan collided, the skull damage to the Chinese human body was more severe, and when a sedan or SUV collided, the brain damage to the Chinese human body was more severe. The existing C-NCAP pedestrian protection testing regulations may not provide the best protection for the Chinese human body. It is recommended that testing regulations be improved by combining collision damage mechanisms and the physical characteristics of Chinese pedestrians.

## Data availability statement

The original contributions presented in the study are included in the article/Supplementary material, further inquiries can be directed to the corresponding authors.

## Author contributions

LY: Writing – original draft, Writing – review & editing, Investigation. CL: Conceptualization, Investigation, Writing – original draft, Writing – review & editing. XZ: Data curation, Methodology, Writing – original draft. DZ: Formal Analysis, Writing – original draft. XL: Methodology, Validation, Writing – original draft. XK: Writing – original draft, Writing – review & editing, Conceptualization, Funding acquisition, Investigation, Resources, Visualization.
