# Supplementary material for: Translational medical bioengineering research of traumatic brain injury among Chinese and American pedestrians caused by vehicle collision based on human body finite element modeling
Source: Front Neurol. 2024 Jan 25;14:1296902. doi: 10.3389/fneur.2023.1296902 (PMC10850393; doi:10.3389/fneur.2023.1296902)
Supplement: Supplementary file 1 [file Table_1.DOCX]

**Supplemental 1**

**The anthropometric data of Post Mortem Human Subjects (PMHS) in Yoganandan's head impact test**

| ID | Sex | Hight(cm) | Body weight (kg) | Lateral-lateral  (cm) | Anteroposterior  (cm) | Nasion-occiput | Circumference | Head Weight（kg） |
| --- | --- | --- | --- | --- | --- | --- | --- | --- |
| 7 | M | 185 | 95 | 15.6 | 18.7 | 21.6 | 16.8 | 4.12 |
| 8 | F | 165 | 68 | 14.0 | 19.1 | 17.5 | 15.9 | 3.21 |
| 9 | F | 168 | 84 | 15.9 | 18.1 | 17.5 | 15.9 | 3.41 |
| 10 | F | 162 | 61 | 14.0 | 18.4 | 17.1 | 14.9 | 3.34 |
| 11 | M | - | - | 14.6 | 19.1 | 19.4 | 14.3 | 3.52 |
| 12 | F | 160 | 36 | 14.7 | 16.5 | 17.0 | 14.5 | 2.81 |
| Mean | - | 168 | 68.8 | 14.8 | 18.3 | 18.4 | 54.6 | 3.7 |

**Supplemental 2**

**The anthropometric data and impact conditions of Post Mortem Human Subjects (PMHS) in Kajzer's lower limb impact test**

| Test  No. | Anthropometric data of PHMS | | | | Impact conditions | | |
| --- | --- | --- | --- | --- | --- | --- | --- |
|  | Sex | Age  (years) | Height  (cm) | Weight  (kg) | Configuration | Leg | Impactor velocity  (km/h) |
| 11B | M | 59 | 170 | 66 | Bending | Right | 39.7 |
| 12S | M | 59 | 170 | 66 | Shearing | Left | 39.5 |
| 13S | M | 44 | 168 | 70 | Shearing | Right | 39.6 |
| 14B | M | 44 | 168 | 70 | Bending | Left | 39.9 |

**Supplemental 3**

**The anthropometric data and impact conditions of Post Mortem Human Subjects (PMHS) in Shaw's thorax impact test**

| Test  No. | Anthropometric data of PHMS | | | | | Impact conditions | | |
| --- | --- | --- | --- | --- | --- | --- | --- | --- |
|  | Sex | Age | Hight(cm) | Mass(kg) | Chest Breadth(cm) | Direction | V(m/s) | Side |
| 0503 | M | 79 | 180 | 65.8 | 30.5 | Lateral | 2.51 | R |
| 0504 | M | 80 | 165 | 80.7 | 32 | Lateral | 2.43 | L |
| 0505 | M | 77 | 178 | 66.2 | 29 | Lateral | 2.45 | R |
| 0506 | M | 87 | 175 | 65.7 | 30.5 | Lateral | 2.55 | R |
| 0507 | M | 53 | 165 | 65.3 | 27.5 | Lateral | 2.48 | L |
| 0601 | M | 63 | 186 | 93 | 30.5 | Lateral | 2.57 | L |
| 0602 | M | 79 | 164 | 74.8 | 30.7 | Lateral | 2.48 | R |
